# Systemic benefits of periodontal therapy in patients with obesity and periodontitis: a systematic review

**DOI:** 10.1590/1807-3107bor-2024.vol38.0031

**Published:** 2024-04-05

**Authors:** Cláudia Callegaro de MENEZES, Davi da Silva BARBIRATO, Mariana Fampa FOGACCI, Guido Artemio MARAÑÓN-VÁSQUEZ, João Régis Ivar CARNEIRO, Lucianne Copple MAIA, Maria Cynésia Medeiros de BARROS

**Affiliations:** (a) Universidade Federal do Rio de Janeiro – UFRJ, Dental School, Division of Periodontics, Rio de Janeiro, RJ, Brazil.; (b) Universidade Federal de Pernambuco – UFPE, Department of Clinical and Preventive Dentistry, Recife, PE, Brazil.; (c) Universidade Federal do Rio de Janeiro – UFRJ, Department of Pediatric Dentistry, Rio de Janeiro, RJ, Brazil.; (d) Universidade Federal do Rio de Janeiro – UFRJ, Clementino Fraga Filho Hospital University, Department of Nutrology/Bariatric Surgery, Rio de Janeiro, RJ, Brazil.; (e) Universidade Federal do Rio de Janeiro – UFRJ, Department of Pediatric Dentistry and Orthodontics, Rio de Janeiro, RJ, Brazil.

**Keywords:** Periodontal diseases, Periodontitis, Obesity, Dental scaling, Root planing

## Abstract

This systematic review aimed to answer the focused question: “What are the benefits of subgingival periodontal therapy on blood hematological and biochemical index, biomarkers of inflammation and oxidative stress, quality of life, and periodontal pathogen counts in patients with obesity and periodontitis?”. A systematic literature search was performed in six databases: PubMed, Embase, LILACS, Web of Science, Cochrane and SCOPUS and other sources, and a manual search was conducted as well. Inclusion criteria were randomized and non-randomized clinical trials, and before-and-after studies on patients with obesity subjected to periodontal therapy. The results were synthesized qualitatively. Risk of bias within studies was assessed using RoB 2 and ROBINS-I tools. The certainty of evidence was evaluated following the GRADE approach. Three randomized controlled trials and 15 before-and-after studies were included. Randomized controlled trials were considered to have a low risk of bias, as compared to before-and-after studies assessed as having low, serious, and critical risks of bias. Non-surgical periodontal therapy plus azithromycin, chlorhexidine, and cetylpyridinium chloride reduced blood pressure and decreased serum levels of HbA1c, hsCRP, IL-1β, and TNF-α. Salivary resistin level also decreased in patients with obesity and periodontitis after therapy and chlorhexidine mouth rinse. Before-and-after data suggest an improvement in total cholesterol, LDL, triglycerides, insulin resistance, C3, GCF levels of TNF-α, chemerin, vaspin, omentin-1, visfatin, 8-OHdG, and periodontal pathogen counts after therapy.

## Introduction

Obesity is known as body mass index (BMI) ≥ 30.0 kg/m^2^, indicating excessive accumulation of fat, which can impair health. It has a high degree of morbidity and it is a risk factor for several types of diseases such as type 2 diabetes mellitus (DM), cardiovascular disease, and cancer.^1-3^ Adipose tissue is a metabolically active endocrine organ^4^ responsible for the increase in serum levels of adipokines in obesity. It is associated with dysregulation of the immunoinflammatory response and endocrine function, hormonal and metabolic disorders, increased susceptibility to infections, hyperinflammatory state, and impaired wound healing. Both obesity and periodontal disease are low-intensity, long-lasting chronic inflammatory diseases, regarded as chronic non-communicable diseases, which share a multifactorial relationship and comorbidities.^5,6^

The interaction between bacterial load and host response links periodontitis to DM, cardiovascular^7,8^ and kidney diseases,^9^ preterm birth, and low birth weight newborn babies.^10^ Although its pathophysiological mechanism is unknown, studies have suggested that obesity may be a risk factor for periodontitis,^11,12^ as first proposed by Perlstein & Bissada.^13^ Some studies propose that the high levels of circulating proinflammatory cytokines such as interleukin-1β (IL-1β), IL-6, tumor necrosis factor-α (TNF-α) in patients with obesity may increase periodontal destruction. ^14,15^ Systematic reviews have evaluated the effect of obesity on non-surgical periodontal therapy (NSPT)^16^ and on periodontal and immunological parameters in patients with obesity, compared to those without obesity.^2,17,18^ Even though periodontal therapy (PT) is associated with reduced periodontal and systemic inflammation in patients with periodontitis and non-communicable diseases with a chronic inflammatory course,^19^ one question remains: Is there evidence that subgingival PT offers systemic benefits for patients with obesity? Accordingly, this review aims to answer the focused question: “What are the benefits of periodontal therapy on blood hematological and biochemical index, biomarkers of inflammation and oxidative stress, quality of life, and periodontal pathogen counts in patients with obesity and periodontitis?”

## Methods

### Protocol and registration

This study was conducted according to the Preferred Reporting Items for Systematic Reviews and Meta-Analyses (PRISMA)^20^. The qualitative synthesis of results followed the SWiM reporting guideline.^21^ Risk of bias within studies were assessed using “Revised Cochrane risk-of-bias tool for randomized trials” (RoB 2), and non-randomized studies of intervention “Risk Of Bias In Non-randomized Studies - of Interventions” (ROBINS-I) tool for (uncontrolled) before-and-after studies. The certainty of evidence was evaluated following the GRADE approach,^22,23^ adapting all the judgments to qualify the evidence in a narrative way.^24^ The review protocol was registered in the PROSPERO (CRD42021241653).

### Search strategy

PubMed, EMBASE, LILACS, Web of Science, Cochrane and SCOPUS databases were systematically searched using the following heading terms: (obesity AND (periodontal diseases OR periodontitis)) AND (root planing OR periodontal therapy OR periodontal treatment OR scaling and root planing).Furthermore, other sources were searched: Google Schoolar, OpenGrey, ClinicalTrials.gov and ReBEC.

### Focused question

Based on the PICO principle—Population: patients with obesity and periodontitis, regardless of age, sex and race; Intervention: periodontal therapy with subgingival approach; Control: no periodontal treatment or supragingival periodontal treatment (without subgingival approach); Outcomes: blood hematological and biochemical index, biomarkers of inflammation and oxidative stress on serum, saliva and gingival crevicular fluid (GCF), quality of life, periodontopathogen counts and adverse effects.

### Study selection criteria

- Inclusion criteria: i- randomized controlled trials (RCTs), non-randomized controlled clinical trials (CCTs) and before-and-after (pre-post) data (BAS) from groups of patients with obesity and periodontitis from clinical trials: ii- studies that evaluated the systemic effect (on serum, saliva and/or gingival crevicular fluid [GCF]) of therapeutic interventions for periodontitis in patients with obesity, with at least a 3-month follow-up; and iii- outcomes of interest.
- Exclusion criteria: i- pilot studies; ii- no description of the periodontitis diagnostic criteria used; iii- participants with congenital syndrome (e.g., Down syndrome, Ehlers-Danlos syndrome, Marfan syndrome, Stickler syndrome, osteogenesis imperfecta, Papillon-Lefevre syndrome, among others.); iv- unavailability of full paper copy; v- trials in which no confirmation or diagnostic criteria for obesity and/or periodontitis were reported and could not be retrieved after contacting the original authors; and vi- trials in which outcomes of interest were not available for analysis and the original values could not be retrieved after contacting the original authors.
- No data or language restrictions were applied.

### Data items and synthesis

Data were independently extracted by two reviewers (blinded process) using a standardized sheet, as recommended by the Cochrane Collaboration’s handbook for systematic review. From the selected articles the following data were extracted: author, country and year; participant’s demographic profile; smoking; alcohol consumption; systemic conditions/diseases, periodontal diagnosis; obesity diagnosis; periods of data collection; characteristics of periodontal intervention; comparison groups; blood hematological and biochemical index; biomarkers of inflammation and oxidative stress; quality of life; study duration (follow-up); periodontal pathogens count and adverse effects. The synthesis of qualitative results followed the SWiM reporting guideline^21^.

### Risk of bias within studies

**Figure 4:**
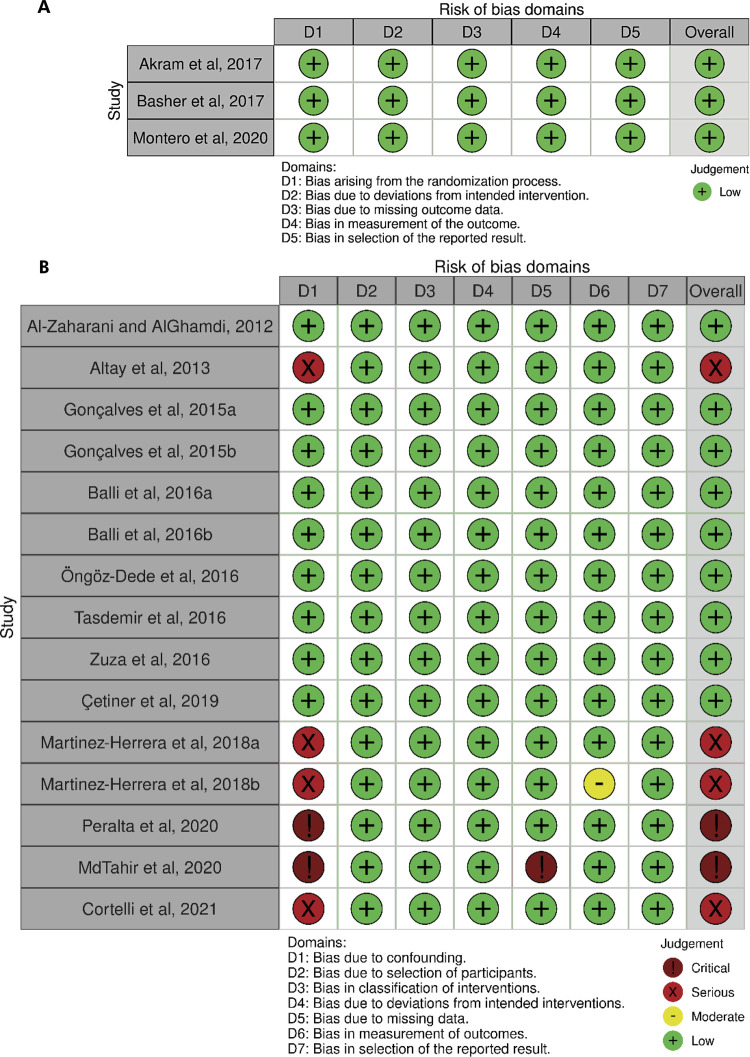
Bias risk analysis dashboard using Cochrane tools: A, “Revised Cochrane risk-of-bias tool for randomized trials” (RoB 2); and B, “Risk Of Bias In Non-randomized Studies - of Interventions” (ROBINS-I) tool for (uncontrolled) before-and-after studies.

The evaluation of quality and risk of bias in clinical studies was performed by two authors independently, using specific risk of bias and methodological quality assessment tools for randomized controlled trials– Figure 4A and 4B.

### Certainty of evidence assessment

The certainty of evidence was evaluated following the GRADE approach^22,23^, adapting all the judgments to qualify the evidence in a narrative way^24^. Thus, the evidence quality index is defined in four categories: high, moderate, low, and very low applied to each of the evaluated outcomes ^22,23^.

## Results

### Study selection

**Figure 1:**
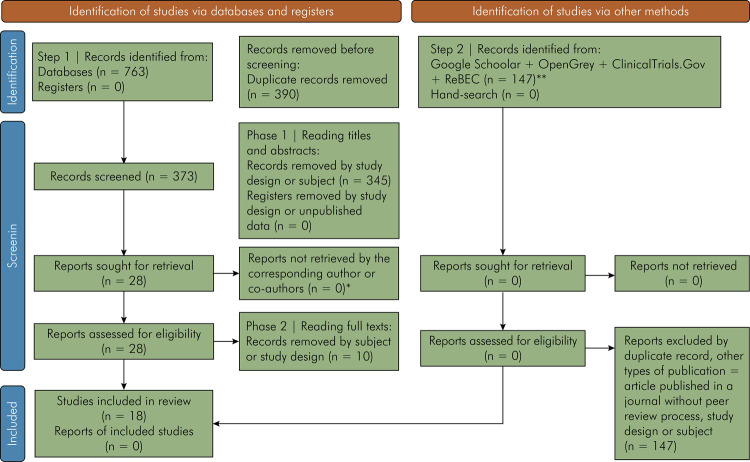
PRISMA flow diagram for new systematic reviews which included searches of databases, registers, and other sources of the screening process. *From:* Page MJ, McKenzie JE, Bossuyt PM, Boutron I, Hoffmann TC, Mulrow CD, et al. The PRISMA 2020 statement: an updated guideline for reporting systematic reviews. BMJ 2021;372:n71. doi: 10.1136/bmj.n71. For more information, visit: http://www.prisma-statement.org/

A total of 763 records were retrieved from the following databases: PubMed (n = 86), Web of Science (n = 101), Cochrane Library (n = 31), Embase (n = 193), Scopus (n = 341), and LILACS (n = 10). After removing 390 duplicates, 345 reports were excluded according to the eligibility criteria, and 28 were selected for full-text reading. Four reports were excluded because of the study design,^25,26,27,28^ five because the obesity group included non-obesity,^29,30,31,32,33^ and one because periodontitis diagnostic criteria were not reported^34^ (Fig. 1). No records were included from the other sources because of subject or duplicity.

Footnote: *, Only Montero et al. (2020) and MdTahir et al. (2020) returned the contact. The other three studies were not excluded, as data available only in graphics were extracted by the WebPlotDigitizer^â^ version 4.4 program; **, the first 100 results of Google Schoolar search results were accessed for the eligibility criteria.

### Study characteristics

A total of 18 reports were included in this systematic review: three RCTs^4,35,36^ and 15 BAS^11,37,38,39,40,41,42,43,44,45,46,47,48,49,50^ (Table 1).

**Table 1:** Descriptive data on medical and periodontal condition.

Author, year	Country/Study design	Elegibility criteria	Participants	Diagnostic criteria		Intervention	Control group	Periodontal maintenance phase | Follow-up
				Obesity	Periodontitis			
Al-Zahrani and AlGhamdi, 2012^11^	Kingdon of Saudi Arabia/BAS	IC: female, ≥ 35 years old, generalized moderate/severe chronic periodontitis and at least 20 remaining teeth	n = 20	BMI ≥ 30 kg/m^2^	≥ 30 % of the sites with CAL ≥ 3 mm	NSPT and OHI	Before NSPT	NA
		EC: systemic diseases or infection, periodontal therapy in the previous 12 months, systemic antibiotic in the previous 3 months, pregnancy or lactation, smokers, antibiotic prophylaxis before periodontal treatment	Mean age: 44. ± 8.4 years					2-months follow-up
Altay et al., 2013^37^	Turkey/BAS	IC: > 25 years old, and ≥ 15 natural remaining teeth	n = 22	BMI ≥ 30 kg/m^2^	≥ 5 teeth with ≥ 1 sites with PD ≥ 5 mm and CAL ≥ 2 mm	FMD	Before NSPT	0.12 % chlorhexidine mouth rinse, b.i.d., for 14 days after NSPT
		EC: antibiotic therapy within the previous 6 months and anti-inflammatory drugs within the previous 3 months, pregnancy or use of contraceptives or any other hormone therapy, periodontal treatment within the previous 24 months, and any systemic problem or treatment during the evaluation period of 3 months before and after periodontal treatment	Males: 5 (22.7 %) Females: 17 (77.3 %)	WC > 102 cm (males) and > 88 cm (females)				Plaque control and OHR at days 1, 7, 14, and 30
			Mean age: 45.6 ± 23.8 years; and Age range: 35–68 years					3-months follow-up
Gonçalves et al., 2015a^38^	Brazil / BAS	IC: > 30 years old, and ≥ 15 remaining teeth excluding third molars and teeth with advanced decay indicated for exodontia, generalized chronic periodontitis, HbA1c < 6.5 %, FPG 70-99 mg/dL, and CRP < 6 mg/L	n = 18	BMI ≥ 30 and < 40 kg/m^2^	> 30 % of the sites with PD and CAL ≥ 4 mm or ≥ 6 teeth with ≥ 1 site with PD and CAL ≥ 5 mm and BOP	NSPT	Before NSPT	Professional plaque control, OHR and SRP of deep sites presenting BOP 3- and 6-months post-therapy
		EC: pregnancy, lactation, current smoking and smoking within the past 10 years, prophylactic antibiotic coverage before dental treatment, subgingival periodontal therapy in the previous 12 months, antimicrobial, anti-inflammatory, immunosuppressive, and lipid-lowering therapies in the previous 6 months, regular use of mouth rinses containing antimicrobials, orthodontic appliances, and presence of systemic conditions that could affect the progression of periodontitis and/or gain/loss of weight	Males: 72.2 % Females: 27.8 %	WHR ≥ 0.9 (males) and ≥ 0.85 (females)				6-months follow-up
			Mean age: 48.8 ± 5.9 years					
Gonçalves et al., 2015b^39^	Brazil / BAS	IC: > 30 years old, and ≥ 15 remaining teeth excluding third molars and teeth with advanced decay indicated for exodontia, generalized chronic periodontitis, HbA1c < 6.5 %, FPG 70-99 mg/dL, and CRP < 6 mg/L	n = 20	BMI ≥ 30 and < 40 kg/m^2^	> 30 % of the sites with PD and CAL ≥ 4 mm or ≥ 6 teeth with ≥ 1 site with PD and CAL ≥ 5 mm and BOP	NSPT	Before NSPT	Periodontal maintenance (non-specified) every 3 months post-therapy
		EC: pregnancy, lactation, current smoking and smoking within the past 10 years, prophylactic antibiotic coverage before dental treatment, subgingival periodontal therapy in the previous 12 months, antimicrobial, anti-inflammatory, immunosuppressive, and lipid-lowering therapies in the previous 6 months, regular use of mouth rinses containing antimicrobials, orthodontic appliances, and presence of systemic conditions that could affect the progression of periodontitis and/or gain/loss of weight	Males: 11 (55 %)	WHR ≥ 0.9 (males) and ≥ 0.85 (females)				12-months follow-up
			Females: 9 (45 %)					
			Mean age: 50 ± 4.5 years					
Balli et al., 2016a	Turkey /BAS	IC: 30–49 years old, > 20 remaining teeth HbA1c < 6.5 %, and FPG < 100 mg/dL	*n* = 20	BMI ≥ 30 and < 40 kg/m^2^	PD and CAL ≥ 5mm with bone loss affecting > 30 % of existing teeth on clinical/radiographic examination	NSPT	Before NSPT	NA
		EC: aggressive periodontitis, periapical pathologies, exposure to mechanical force as a result of occlusion/orthodontics, systemic diseases such as cancer, HIV, diabetes mellitus or additional diseases which may interfere with adipokines levels and the periodontal conditions high-grade steroid therapies, radiation/immuno-suppressive therapies, pregnancy, lactation, smoking over the past five years, allergic reaction to any kind of drug, no history of either periodontal or drug therapies within the preceding six months, namely anti-inflammatory treatments, and antibiotic courses or other pharmacological treatments	Males: 9 (45 %)	WHR ≥ 0.9 (males) and ≥ 0.85 (females)				6-weeks follow-up
			Females: 11 (55 %)					
			Mean age: 40.56 ± 4.11 years					
Balli et al., 2016a	Turkey /BAS	IC: 30–49 years old, > 20 remaining teeth HbA1c < 6.5 %, and FPG < 100 mg/dL	*n* = 20	BMI ≥ 30 and < 40 kg/m^2^	PD and CAL ≥ 5mm with bone loss affecting > 30 % of existing teeth on clinical/radiographic examination	NSPT	Before NSPT	NA
		EC: aggressive periodontitis, periapical pathologies, exposure to mechanical force as a result of occlusion/orthodontics, systemic diseases such as cancer, HIV, diabetes mellitus or additional diseases which may interfere with adipokines levels and the periodontal conditions high-grade steroid therapies, radiation/immuno-suppressive therapies, pregnancy, lactation, smoking over the past five years, allergic reaction to any kind of drug, no history of either periodontal or drug therapies within the preceding six months, namely anti-inflammatory treatments, and antibiotic courses or other pharmacological treatments	Males: 9 (45 %)	WHR ≥ 0.9 (males) and ≥ 0.85 (females)				6-weeks follow-up
			Females: 11 (55 %)					
			Age: 42 (36–46) years					
Öngöz Dede et al., 2016b^42^	Turkey /BAS	IC: ≥ 20 remaining teeth excluding third molars, non-smokers who had never smoked, no history of systemic disease, had not undergone periodontal therapy or taken medicine for at least 6 months before the study, no pregnancy or lactation, and no alcohol or antioxidant vitamin consumption	*n* = 15	BMI ≥ 30 kg/m^2^	PD and CAL ≥ 5mm with bone loss affecting > 30 % of existing teeth on clinical/radiographic examination	Intensive hygiene phase and full-mouth NSPT, and the maintenance and monitoring of oral hygiene	Before NSPT	Periodontal maintenance and monitoring of oral hygiene (non-specified)
			Males: 8 (53.3 %)					4-weeks follow-up
			Females: 7 (46.7 %)					
			Mean age: 47.13 ± 7.17 years; and Age range: 34–60 years					
Taşdemir et al., 2016^43^	Turkey /BAS	IC: > 25 years old, chronic periodontitis, obesity, ≥ 15 remaining teeth, and type 2 diabetes mellitus (in diabetes group)	*n* = 14	BMI ≥ 30 kg/m^2^	≥ 5 teeth with ≥ 1 sites with PD ≥ 5 mm and CAL ≥ 2 mm	Intensive full-mouth NSPT	Before FMT	NA
		EC: antibiotic or anti-inflammatory drug use within the previous 6 months, pregnancy or lactation, periodontal therapy within the previous 6 months, smoking or history of smoking, alcohol consumption, and lipid lowering medications	Males: 9 (64.3 %) Females: 5 (35.7 %)	WC > 102 cm (males) and > 88 cm (females)				6-months follow-up
			Mean age: 49.2 ± 9.2; and Age range: 30–62 years					
Zuza et al., 2016^44^	Brazil / BAS	IC: 35–55 years old, both sexes, chronic periodontitis, and ≥ 20 remaining teeth	*n* = 28	BMI ≥ 30 and < 40 kg/m^2^	≥ 6 teeth with PD ≥5 mm and CAL ≥ 3 mm and BOP	NSPT and OHI and motivation	Before NSPT	3-months follow-up
		EC: smokers or former smokers, antibiotics or anti-inflammatory in prior 3 months, diabetes, or other systemic diseases, pregnant or lactating women, use of hormones, mental or physical limitations, and periodontal therapy in the previous 12 months	Males: 6 (21.4 %)	WHR ≥ 0.9 (males) and ≥ 0.85 (females)				
			Females: 22 (78.6 %)	WC >102 cm (male) and >88 cm (female)				
			Mean age: 45.7 ± 8.4 years	% of body fat ≥25% (male) ≥35% (female)				
Akram et al., 2017 ^67^	Malaysia/RCT	IC: 30–66 years old, chronic periodontitis, and ≥ 12 remaining teeth excluding third molars	*n* = 62	BMI ≥ 27.5 kg/m^2^	≥ 2 interproximal sites with PD ≥ 5 mm (different teeth) or ≥ 2 interproximal sites with CAL ≥ 4 mm (different teeth)	NSPT, OHI and 0.12 % chlorhexidine mouth rinse	No periodontal therapy or oral hygiene instruction	Professional prophylaxis, re-motivation and OHR
		EC: pregnant or lactating mothers medical condition requiring prophylactic antibiotic administration before dental treatment, periodontal treatment during the previous 6 months, intellectual disability that might interfere with oral hygiene procedures, not Malaysian, presence of systemic conditions that could affect progression of periodontitis, or weight gain/loss or other inflammatory conditions	CG = 31 and IG = 31	WHR ≥ 0.9 (males) and ≥ 0.85 (females)				6-weeks and 3-months follow-up
			Males: 17 (27.4 %); CG = 9 (32.3 %) and IG = 8 (25.8 %)					
			Females: 45 (72.6 %); CG = 22 (67.7 %) and IG = 23 (74.2 %)					
			Mean age: CG = 44.84 ± 9.02 years and IG = 44.68 ± 10.63 years					
Basher et al., 2017^35^	Malaysia/RCT	IC: malaysians, ≥ 30 years old, chronic periodontitis, obesity, and ≥ 12 remaining teeth	*n* = 62	BMI ≥ 27.5 kg/m^2^	≥ 2 interproximal sites with PD ≥ 4 mm and ≥ 2 interproximal sites with CAL ≥ 3 mm (different teeth) or one site with PD ≥ 5 mm	NSPT, OHI and 0.12 % chlorhexidine mouth rinse	No periodontal therapy or oral hygiene instruction	Professional prophylaxis, re-motivation and OHR
		EC: periodontal treatment within the past 6 months, antibiotic treatment within the past 4 months, require prophylactic antibiotic coverage, use of systemic or topical NSAIDs for the past 4 months, pregnant or intend to and lactating mothers, mentally handicapped, rheumatic heart disease, and valve replacement	CG = 31 and IG = 31					3-months follow-up
			Males: 18 (29 %); CG = 10 (32.25 %) and IG = 8 (25.8 %)					
			Females: 44 (71 %); CG = 21 (67.75 %) and IG = 23 (74.20 %)					
			Mean age: CG = 44.85 ± 9.02 years and IG = 45.03 ± 10.72 years					
Martínez-Herrera et al., 2018a^46^	Spain/BAS	EC: aggressive periodontitis, < 14 remaining teeth, infectious or other inflammatory diseases, periodontal therapy in the last 6 months or antibiotics in the last 3 months, treatment with systemic anti-inflammatory drugs, pregnancy or lactation, secondary obesity, antibiotic treatment before the dental intervention, and diabetes mellitus	At baseline, *n* = 96	BMI ≥ 30 kg/m^2^	≥ 4 teeth with ≥1 sites with PD ≥ 4 mm and CAL ≥ 3 mm	Intensive full-mouth NSPT, OHI and 0.12 % chlorhexidine mouth rinse	Before NSPT	Periodontal examinations at 3 months post-therapy
			Males: 29 %					3-months follow-up
			Females: 71 %					
			Mean age: 42.7 ± 10.2 years					
			After 3 months, *n* = 74					
Martínez-Herrera et al., 2018b^47^	Spain/BAS	EC: aggressive periodontitis, < 14 remaining teeth, infectious or other inflammatory diseases, periodontal therapy in the last 6 months or antibiotics in the last 3 months, treatment with systemic anti-inflammatory drugs, pregnancy or lactation, secondary obesity, antibiotic treatment before the dental intervention, and diabetes mellitus	*n* = 47	BMI ≥ 30 kg/m^2^	≥ 4 teeth with ≥ 1 sites with PD ≥ 4 mm and CAL ≥ 3 mm	Intensive full-mouth NSPT, OHI and 0.12 % chlorhexidine mouth rinse	Before NSPT	Periodontal examinations at 3-months post-therapy
			Males: 31.9 %					3-months follow-up
			Females: 68.1 %					
			Mean age: 44.4 ± 10.4 years					
Çetiner et al., 2019^45^	Turkey /BAS	IC: > 20 years old, > 22 remaining teeth, and no systemic diseases	*n* = 21	BMI ≥ 30 kg/m^2^	≥ 30 % of the sites with bone loss and ≥ 2 non-adjacent teeth with ≥ 1 sites with PD ≥ 5 mm and CAL ≥ 5 mm in each quadrant and BOP	Intensive full-mouth NSPT	Before FMT	NA
		EC: localized chronic periodontitis, receiving periodontal therapy/surgery in the last 6 months, pregnancy or use of any hormone therapy, antibiotic or anti-inflammatory drug therapy within the last 6 months, smoker, lactating, aggressive periodontitis, and periapical pathologies	Females: 100 %	WC > 102 cm (males) and > 88 cm (females)				3-months follow-up
			Mean age: 44.67 ± 10.87 years					
Peralta et al., 2020^48^	Brazil / BAS	IC: ≥ 45 years old, both sexes, moderate, severe, and advanced periodontitis, and ≥ 12 remaining teeth	*n* = 55	BMI ≥ 30 kg/m^2^	Stage II: interdental PD ≤ 5 mm, CAL 3 to 4 mm, and radiographic bone loss at coronal third between 15 % to 33 %	FMD	Before NSPT	0.12 % chlorhexidine mouth rinse for 14 days post-therapy
		EC: orthodontic devices, pregnancy or breast-feeding, systemic diseases or other conditions that could influence the periodontal status (other than diabetes), alcohol abuse, prophylactic antibiotic coverage, systemic antibiotics and/or anti-inflammatory drugs six months prior to the study, and periodontal therapy within six months prior to the study	Males: 19 (34.5 %)	WC > 102 cm (males) and > 88 cm (females)	Stage III and IV: PD ≥ 6 mm, interdental CAL ≥ 5 mm, and radiographic bone loss extending to mild-third of the root			Every 3-months, OHR, supragingival dental scaling and professional prophylaxis
			Females: 36 (65.5 %)					
			Mean age: 48.9 ± 7.8 years					
Md Tahir et al., 2020^49^	Malaysia/BAS	IC: > 30 years old, obesity and normal weight, and ≥ 12 remaining teeth	*n* = 18	BMI ≥ 30 kg/m^2^	≥ 2 interproximal sites with PD ≥ 4 mm and ≥ 2 interproximal sites with CAL ≥ 3 mm (different teeth) or one site with PD ≥ 5 mm	Intensive full-mouth NSPT and OHI	Before NSPT	15 mL 0.12 % chlorhexidine mouth rinse, t.i.d., for 14 days post-therapy
		EC: history of periodontal therapy in last 6 months, on antibiotics and topical/systemic steroid treatment in last 4 months, pregnancy, lactating mothers, mentally handicapped, and valve replacement and rheumatic heart disease which require antibiotic coverage	Males: 6 (33.3 %)			Root surface debridement at sites with PD ≥ 5mm		3-months follow-up
			Females: 12 (66.7 %)			Periodontal pockets irrigated with 0.12 % chlorhexidine		
			Mean age: 44.7 ± 2.4 years			0.12 % chlorhexidine mouth rinse		
Montero et al., 2020^36^	Spain/RCT	IC: 35–65 years old, metabolic syndrome [MetS (at least, 3 risk factors: WC ≥ 94 cm in men and ≥ 80 cm in women, triglycerides ≥150 mg/dL, HDL < 40 mg/dL in males and < 50 mg/dL in females, BP systolic ≥ 130 and/or diastolic ≥ 85 mm Hg, FPG ≥ 100 mg/dL)], stages III-IV generalized periodontitis, and ≥ 16 remaining teeth	*n* = 63	WC ≥ 94 cm (males) and ≥ 80 cm (females)	≥ 8 sites with PD ≥ 6 mm and 4 sites with CAL ≥ 5 mm in ≥ 2 different quadrants	NSPT, OHI and administration of a systemic antibiotic (azithromycin 500 mg, q.d., for 3 days), administered at the last session of SRP	Minimal periodontal therapy (supragingival professional mechanical plaque and calculus removal) + administration of placebo medication for 3 days + OHI	0.12 % chlorhexidine and 0.05 % cetylpyridinium chloride mouth rinse for 14 days post-therapy
		EC: uncontrolled systemic diseases other than diabetes or hypertension, surgical treatment in the previous 3 months, alcoholism or psychiatric disorders, systemic antibiotic in the previous 3 months, NSPT in the previous 6 months, or surgical periodontal treatment over the previous 12 months	CG = 31 and IG = 32					professional prophylaxis in both groups at the 3- and 6-months post-therapy
			Males: 44 (69.8 %); CG = 22 (70.9 %) and IG = 22 (68.8 %)					6-months follow-up
			Females: 19 (30.2 %); CG = 9 (29.1 %) and IG = 10 (31.2 %)					
			Mean age: CG = 58.3 ± 5.8 years and IG = 56.7 ± 6.5 years					
Cortelli et al., 2021^50^	Brazil / BAS	IC: ≥ 45 years old, both genders, moderate to advanced generalized periodontitis (Stage II-IV), and ≥ 12 remaining teeth	*n* = 55	BMI ≥ 30 kg/m^2^	Interproximal CAL detectable in ≥ 2 teeth (non-adjacent)	FMD	Before NSPT	0.12 % chlorhexidine mouth rinse for 14 days post-therapy
		EC: chronic renal failure, stroke history, not controlled diabetes, rheumatism, osteoporosis, HIV, acute myocardial infarction 6 months before the study, pregnant and lactating, and periodontal treatment in last year	Males: 19 (34.5 %)	WC > 102 cm (males) and > 88 cm (females)	or			Every 3 months, OHR, professional prophylaxis and supragingival debridement
			Females: 36 (65.5 %)		PD > 3 mm and CAL ≥ 3 mm in ≥ 2 teeth			6-months follow-up
			Mean age: 48.9 ± 7.8 years					

RCT, randomized controlled trial; BAS, before and after (pre-post) study; IC, inclusion criteria; EC, exclusion criteria; CG, control group; IG, intervention group; NSAID, non-steroidal anti-inflammatory drugs; PD, probing depth; CAL, clinical attachment level/loss; BOP, bleeding on probing; HDL, high density lipoprotein cholesterol; BP, blood pressure; FPG, fasting plasma glucose; HbA1c, glycated hemoglobin; FPG, fasting plasma glucose; CRP, C-reactive protein; n, sample size; Age, mean (standard deviation) or median (percentile 25–75); BMI, body mass index; WC, waist circumference; WHR, waist-hip ratio; NSPT, non-surgical periodontal therapy (supragingival plaque and calculus removal and subgingival scaling and root planning); FMD, full-mouth disinfection protocol adapted from Quirynen et al. (1995); OHI, oral hygiene instructions; OHR, oral hygiene reinstructions; NA, data not available.

**Figure 2:**
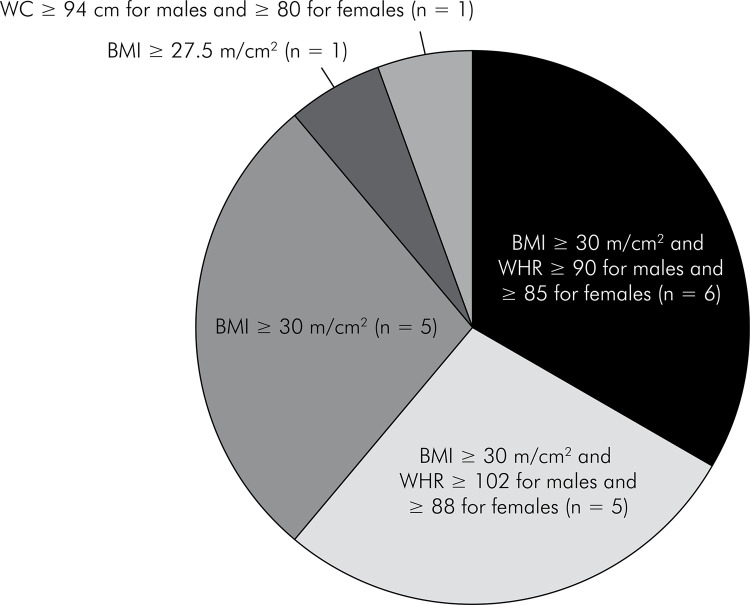
Descriptive pie chart of diagnostic criteria for obesity reported in the studies.

**Figure 3:**
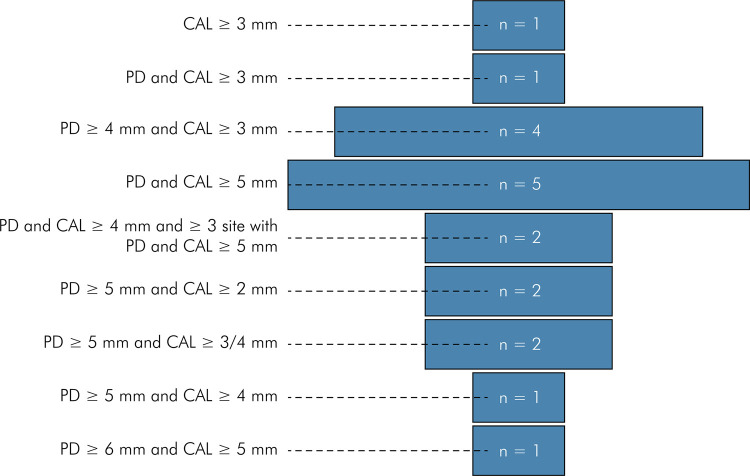
Descriptive funnel chart of diagnostic criteria for periodontitis reported in the studies.

A total of 634 patients with obesity and periodontitis were considered for analysis, among whom 187 were from RCTs and 447 from BAS. The diagnostic criteria for obesity and periodontitis varied between studies, as reported in Table 1 and Figures 2 and 3. The distribution of smokers in the control group (CG) and intervention group (IG) did not differ between the three RCTs.^4,35,36^ Akram et al.^4^ controlled statistical analyses for the assessment of smoking and Montero et al.^36^ reported adjusted p-values for this variable.

Footnotes: BMI, bone mass index (m/cm^2^); WC, waist circumference (cm); WHR, waist-to-hip ratio. Six studies used body mass index (BMI) ≥ 30 m/cm^2^ and waist-hip ratio (WHR) ≥ 0.9 for males ≥ 0.85 for females (Gonçalves et al., 2015a and 2015b; Akram et al., 2016; Balli et al., 2016a and 2016b; and Zuza et al., 2016); five studies used BMI ≥ 30m/cm^2^ and waist circumference (WC) > 102 cm for males and > 88 cm for females (Altay et al., 2013; Taşdemir et al., 2016; Çetiner et al., 2019; Peralta et al., 2020; Cortelli et al., 2021); five reports used BMI ≥ 30 m/cm^2^ (Al-Zaharani and AlGhamdi, 2012; Öngöz-Dede et al., 2016; Martinez-Herrera et al., 2018a and 2018b; MdTahir et al., 2020), one study used BMI ≥ 27.5m/cm^2^ (Basher et al., 2017), and one study used WC ≥ 94 cm in males and ≥ 80 cm in females (Montero et al., 2020).

Footnote: *n*, absolute frequency of studies per criteria; CAL, clinical attachment level (mm); PD, probing depth (mm). The limit score of clinical attachment level (CAL) and probing depth (PD) varied between studies. Four studies used PD ≥ 4 mm and CAL ≥ 3 mm (Martinez-Herrera et al., 2015a, 2015b; Basher et al., 2017; MdTahir et al., 2020); four studies used PD and CAL ≥ 5 mm (Balli et al., 2016a, 2016b; Öngöz-Dede et al., 2016; Çetiner et al., 2019); two studies used PD and CAL ≥ 4 mm and ≥ 1 site with PD and CAL ≥ 5 mm (Gonçalves et al., 2015a, 2015b); two studies used PD ≥ 5 mm and CAL ≥ 3/4 mm (Zuza et al., 2016; Peralta et al., 2020); and two studies used PD ≥ 5 mm and CAL ≥ 2 mm (Altay et al., 2013; Taşdemir et al., 2016). Other scores were used by one report only: CAL ≥ 3 mm (Al-Zaharani and AlGhamdi, 2012); PD ≥ 5 mm and CAL ≥ 4 mm (Akram et al., 2016); PD ≥ 6 mm and CAL ≥ 5 mm (Montero et al., 2020); and PD and CAL ≥ 3mm (Cortelli et al., 2021)

Among BAS studies, all participants with obesity and periodontitis were evaluated before and after PT: seven studies performed NSPT in more than one session;^11,38,39,40,41,44^ six performed intensive full-mouth NSPT,^42,43,45,46,47,49^ and three^37,48,50^ adopted the full-mouth disinfection protocol proposed by Quirynen et al.^51^ Chlorhexidine protocols adjuvant to NSPT and in the periodontal maintenance phase varied between studies (Table 1). BAS data from five studies^37,46,47,48,49^ included smokers.

### Results of individual studies

Individual descriptive data from included studies are presented in Table 2 for RCT outcomes, and Table 3 for BAS outcomes.

**Table 2:** Primary outcome measures for RCT studies.

Object of investigation	Follow-up	Akram et al., 2017^67^		Basher et al., 2017^35^		Montero et al., 2020^36^	
		CG (*n=*31)	IG (*n=*31)	CG (*n=*31)	IG (*n=*31)	CG (*n*=31)	IG (*n=*32)
Blood hematological and biochemical index							
High-sensitivity C-reactive protein (hsCRP) - mg/L	Baseline	-	-	-	-	3.9 ± 3.4^A^	3.9 ± 2.9^A^
	3 months	-	-	-	-	3.9 ± 0.6^B^	2.7 ± 0.4^B^
	6 months	-	-	-	-	4 ± 0.8^B^	2.9 ± 0.4^B^
Fibrinogen - mg/dL	Baseline	-	-	-	-	398.5 ± 89.1^A^	419.7 ± 108.7^A^
	3 months	-	-	-	-	398.3 ± 17.9^B^	421.8 ± 20.4^B^
	6 months	-	-	-	-	400.5 ± 16.1^B^	419.6 ± 21.8^B^
White blood cells count - K/µL	Before	-	-	-	-	7.5 ± 1.7^A^	7.8 ± 1.9^A^
	3 months	-	-	-	-	7.8 ± 0.3^B^	7.5 ± 0.4^B^
	6 months	-	-	-	-	7.6 ± 0.2^B^	7.9 ± 0.7^B^
Glycated hemoglobin (Hba1c) - %	Before	-	-	-	-	6 ± 1^A^	6.3 ± 1.2^A^
	3 months	-	-	-	-	6.1 ± 0.2^A^	5.9 ± 0.1^A^
	6 months	-	-	-	-	6.1 ± 0.2^A^	6 ± 0.1^A^
Fasting plasma glucose - mg/dL	Before	-	-	-	-	133 ± 51.7^A^	128.6 ± 30.3^A^
	3 months	-	-	-	-	130 ± 8.8^B^	123.3 ± 7.9^B^
	6 months	-	-	-	-	130.5 ± 9.7^B^	121 ± 6.3^B^
Fasting insulin - mIU/L	Before	-	-	-	-	14.5 ± 9.3^A^	19.3 ± 10.8^A^
	3 months	-	-	-	-	14.1 ± 1.4^B^	17.2 ± 2.9^B^
	6 months	-	-	-	-	14.4 ± 1.7^B^	14.3 ± 2.1^B^
Total cholesterol - mg/dL	Before	-	-	-	-	189.4 ± 48.4^A^	174.8 ± 34.7^A^
	3 months	-	-	-	-	180.6 ± 8.1^B^	184 ± 8.4^B^
	6 months	-	-	-	-	189.9 ± 9.2^B^	183.5 ± 7.5^B^
High density lipoprotein cholesterol (HDL) - mg/dL	Before	-	-	-	-	46.9 ± 12.4^A^	46.1 ± 13.3^A^
	3 months	-	-	-	-	47.1 ± 3.1^B^	46.2 ± 3.8^B^
	6 months	-	-	-	-	48.4 ± 2.7^B^	47.2 ± 2.7^B^
Low density lipoprotein cholesterol (LDL) - mg/dL	Before	-	-	-	-	105.7 ± 44.9^A^	114.3 ± 34.7^A^
	3 months	-	-	-	-	103.5 ± 7^B^	109.6 ± 8.5^B^
	6 months	-	-	-	-	107.5 ± 8.3^B^	107.6 ± 6.6^B^
Triglycerides - mg/dL	Before	-	-	-	-	136.6 ± 42.5^A^	129.5 ± 52.3^A^
	3 months	-	-	-	-	155.4 ± 17.5^B^	136.5 ± 9.7^B^
	6 months	-	-	-	-	131.7 ± 8.3^B^	125.6 ± 9.7^B^
Creatinine - mg/dL	Before	-	-	-	-	0.9 ± 0.3^A^	0.9 ± 0.5^A^
	3 months	-	-	-	-	0.9 ± 0.1^B^	1.0 ± 0.1^B^
	6 months	-	-	-	-	1 ± 0.1^B^	1.0 ± 0.1^B^
α-1 antitrypsin - mg/dL	Before	-	-	-	-	138.5 ± 28.1^A^	145.6 ± 29.7^A^
	3 months	-	-	-	-	130 ± 5^B^	138.4 ± 6.2^B^
	6 months	-	-	-	-	127.6 ± 5.2^B^	137.5 ± 5.7^B^
Homeostatic model assessment 2 (HOMA2) β-cell function	Before	-	-	-	-	92.7 ± 50.6^A^	104.8 ± 69^A^
	3 months	-	-	-	-	87.2 ± 10.6^B^	106 ± 14.8^B^
	6 months	-	-	-	-	100.4 ± 11.9^B^	106.1 ± 14^B^
Homeostatic model assessment 2 (HOMA2) insulin sensitivity	Before	-	-	-	-	62.6 ± 28^A^	59 ± 55.3^A^
	3 months	-	-	-	-	57.9 ± 5.4^B^	67 ± 13.6^B^
	6 months	-	-	-	-	59.7 ± 5.3^B^	65.8 ± 11.6^B^
Homeostatic model assessment 2 (HOMA2) insulin resistance	Before	-	-	-	-	2 ± 1.2^A^	2.6 ± 1.4^A^
	3 months	-	-	-	-	2 ± 0.2^B^	2.3 ± 0.4^B^
	6 months	-	-	-	-	2 ± 0.2^B^	2.2 ± 0.3^B^
Systemic biomarkers of inflammation							
Resistin - ng/mL (blood serum)	Baseline	14.25 ± 4.58^A^	12.26 ± 1.24^A^	-	-	-	-
	3 months	13.47 ± 5.20^A^	11.62 ± 0.90^A^	-	-	-	-
Interlukin-1β (IL-1β) - pg/mL (blood serum)	Baseline	-	-	-	-	1.9 ± 1.2^A^	1.5 ± 0.9^A^
	3 months	-	-	-	-	2.3 ± 0.5^B^	0.9 ± 0.1^B^
	6 months	-	-	-	-	1.5 ± 0.2^B^	1.5 ± 0.2^B^
Interleukin-6 (IL-6) - pg/mL (blood serum)	Baseline	-	-	-	-	2.8 ± 1.9^A^	2.2 ± 1.8^A^
	3 months	-	-	-	-	2.6 ± 0.4^B^	1.9 ± 0.4^B^
	6 months	-	-	-	-	2.5 ± 0.4^B^	2.0 ± 0.4^B^
Interleukin-8 (IL-8) - pg/mL (blood serum)	Baseline	-	-	-	-	5.4 ± 3^A^	6.9 ± 9.7^A^
	3 months	-	-	-	-	5.4 ± 0.8^B^	4.6 ± 1.1^B^
	6 months	-	-	-	-	6 ± 1.2^B^	5 ± 1.2^B^
Tumor necrosis factor-α (TNF-α) - pg/mL (blood serum)	Baseline	-	-	-	-	8.7 ± 8.6^A^	7.9 ± 6.2^A^
	3 months	-	-	-	-	10 ± 2.3^B^	6.4 ± 0.8^B^
	6 months	-	-	-	-	8.2 ± 1.4^B^	6.3 ± 0.8^B^
Quality of Life							
Oral Health Impact Profile (OHIP PI)	Baseline	-	-	19 ± 61.29^A^	21 ± 67.74^A^	-	-
	3 months	-	-	12 ± 38.71^A^	10 ± 32.26^A^	-	-
Oral Health Impact Profile (OHIP SS)	Baseline	-	-	58.29 ± 6.12^A^	57.2 ± 8.61^A^	-	-
	3 months	-	-	60.95 ± 6.64^A^	61.89 ± 7.04^A^	-	-
Oral Health Impact Profile (OHIP EI)	Baseline	-	-	1.5 ± 1.53^A^	1.62 ± 1.84^A^	-	-
	3 months	-	-	0.65 ± 1.02^A^	0.47 ± 0.91^A^	-	-
Periodontal pathogens count/group							
*Porphyromonas gingivalis* (log of CFU)	Baseline	-	-	-	-	13.3 ± 2.1^A^	11.3 ± 5.7^A^
	3 months	-	-	-	-	11.8 ± 1^B^	3.8 ± 0.9^B^
	6 months	-	-	-	-	11.8 ± 1.1^B^	4.5 ± 1^B^
*Prevotella intermedia* (log of CFU)	Baseline	-	-	-	-	11.2 ± 3.7^A^	10.3 ± 5.1^A^
	3 months	-	-	-	-	10.3 ± 1^B^	4.2 ± 1^B^
	6 months	-	-	-	-	9.9 ± 1^B^	6.2 ± 1^B^
*Aggregatibacter actinomycetemcomitans* (log of CFU)	Baseline	-	-	-	-	0	0.9 ± 2.8^A^
	3 months	-	-	-	-	0.4 ± 0.4^B^	0
	6 months	-	-	-	-	0	0.1 ± 0.1^B^
*Tannerella forsythia* (log of CFU)	Baseline	-	-	-	-	6.3 ± 6.1^A^	6.7 ± 6.3^A^
	3 months	-	-	-	-	6.1 ± 1.3^B^	0.3 ± 0.3^B^
	6 months	-	-	-	-	5.2 ± 1.3^B^	2.4 ± 0.8^B^
*Parvimonas micra* (log of CFU)	Baseline	-	-	-	-	1.3 ± 3.4^A^	2 ± 4.6^A^
	3 months	-	-	-	-	1.8 ± 0.9^B^	1.4 ± 0.7^B^
	6 months	-	-	-	-	0.7 ± 0.5^B^	0.3 ± 0.3^B^
*Fusobacterium nucleatum* (log of CFU)	Baseline	-	-	-	-	10 ± 4.1^A^	8 ± 5.6^A^
	3 months	-	-	-	-	9.4 ± 1.1^B^	6.4 ± 0.9^B^
	6 months	-	-	-	-	8.3 ± 1.2^B^	6.1 ± 0.9^B^
*Campylobacter rectus* (log of CFU)	Baseline	-	-	-	-	NA	1.1 ± 3.4^A^
	3 months	-	-	-	-	0.9 ± 0.6^B^	0.7 ± 0.5^B^
	6 months	-	-	-	-	0	1.0 ± 0^B^
*Eikenella corrodens* (log of CFU)	Baseline	-	-	-	-	2.4 ± 4.7^A^	1.9 ± 4.2^A^
	3 months	-	-	-	-	2.4 ± 0.9^B^	1.2 ± 0.6^B^
	6 months	-	-	-	-	3.3 ± 1^B^	1.2 ± 0.6^B^
*Capnocytophaga* spp. (log of CFU)	Baseline	-	-	-	-	1.7 ± 4.1^A^	1.3 ± 3.5^A^
	3 months	-	-	-	-	2 ± 0.8^B^	1 ± 0.6^B^
	6 months	-	-	-	-	0.4 ± 0.4^B^	1.2 ± 0.6^B^

CG, control group; IG, intervention group; *n*, sample size; OHIP-14 (Oral Health Impact Profile-14): PI_ prevalence of impact, SS_ severity score, and EI_ extent of impact; CFU, colony-forming units; ^A^, mean ± standard deviation; ^B^, mean ± standard error; -, variable not assessed by the authors; NA, data not available.

**Table 3:** Descriptive data on primary outcomes for BAS.

Object of investigation	Follow-up	Al-Zahrani and AlGhamdi, 2012^11^	Altay et al., 2013^37^	Gonçalves et al., 2015a^38^	Gonçalves et al., 2015b^39^	Balli et al., 2016a^40^	Balli et al., 2016b^41^	Öngöz-Dede et al., 2016^42^
		( = 20)	( = 22)	( = 18)	( = 20)	( = 20)	( = 20)	( = 15)
**Blood hematological and biochemical index**								
High-sensitivity C-reactive protein (hsCRP) - mg/L	Baseline	0.96 ± 0.41^A^	3.3 (3.2 to 6)^C^	-	-	-	-	-
	2 months	D = 0.19 ± 0.32^A^	-	-	-	-	-	-
	3 months	-	3 (3.1 to 4.1)^C^	-	-	-	-	-
Fasting blood glucose - mg/dL	Before	-	104 (93 to 115)^C^	-	-	-	-	-
	3 months	-	97 (83 to 109)^C^	-	-	-	-	-
Insulin - µU/mL	Before	-	16.8 (11.5 to 24.8)^C^	-	-	-	-	-
	3 months	-	15.1 (7.1 to 17.8)^C^	-	-	-	-	-
Homeostasis model assessment of insulin resistance (HOMA-IR)	Before	-	4.9 (1.1 to 11.8)^C^	-	-	-	-	-
	3 months	-	3.6 (0.79 to 7.8)^C^	-	-	-	-	-
Total cholesterol - mg/dL	Before	-	194 ± 37^A^	-	-	-	-	-
	3 months	-	188 ± 31^A^	-	-	-	-	-
High density lipoprotein cholesterol (HDL) - mg/dL	Before	-	41 ± 9^A^	-	-	-	-	-
	3 months	-	41 ± 7^A^	-	-	-	-	-
Low density lipoprotein cholesterol (LDL) - mg/dL	Before	-	107 (96 to 134)^C^	-	-	-	-	-
	3 months	-	103 (91 to 128)^C^	-	-	-	-	-
Triglycerides - mg/dL	Before	-	167 (135 to 224)^C^	-	-	-	-	-
	3 months	-	162 (113 to 202)^C^	-	-	-	-	-
Lipoprotein-a - g/L	Baseline	-	0.15 (0.1 to 0.22)^C^	-	-	-	-	-
	3 months	-	0.14 (0.1 to 0.2)^C^	-	-	-	-	-
**Systemic biomarkers of inflammation**								
Tumor necrosis factor-α (TNF-α) - mg/L^¢^ or pg/mL^¥^ (blood serum)	Before	-	5.4 (3 to 9.1)^C¢^	-	3 ± 0.8^A¥^	-	-	-
	3 months	-	3.3 (2.8 to 5.5)^C¢^	-	3.1 ± 1.4^A¥^	-	-	-
	6 months	-	-	-	3.1 ± 1^A¥^	-	-	-
	12 months	-	-	-	2.9 ± 1^A¥^	-	-	-
Tumor necrosis factor-α (TNF-α) - pg/mL (gingival crevicular fluid)	Baseline	-	-	-	-	-	10.8 (6.2 up to 14.2)^D^	-
	6 weeks	-	-	-	-	-	3.8 (3.1 up to 4.8)^D^	-
Interleukin-6 (IL-6) - ng/L^£^ or pg/mL^¥^ (blood serum)	Before	-	1.1 (0.8 to 1.9)^C£^	-	2.7 ± 1.6^A¥^		-	-
	3 months	-	0.6 (0.3 to 1.4)^C£^	-	2.9 ± 0.9^A¥^	-	-	-
	6 months	-	-	-	2.3 ± 0.8^A¥^	-	-	-
	12 months	-	-	-	2.3 ± 0.8^A¥^	-	-	-
Interleukin-6 (IL-6) - pg/mL (gingival crevicular fluid)	Baseline	-	-	-	-	2.3 (2 up to 2.7)^D^	-	-
	6 weeks	-	-	-	-	0.7 (0.3 up to 1.2)^D^	-	-
Resistin - ng/mL x 5 (blood serum)	Before	-	-	-	2.9 ± 1.7^A^	-	-	-
	3 months	-	-	-	3.3 ± 1.7^A^	-	-	-
	6 months	-	-	-	3.3 ± 2^A^	-	-	-
	12 months	-	-	-	3.2 ± 2.3^A^	-	-	-
Leptin - ng/L^£^ or pg/mL^¥^ x 100 (blood serum)	Baseline	-	17.5 (4.3 to 43.9)^C£^	441.8 ± 213.7^A¥^	481.8 ± 415.5^A¥^	-	-	-
	3 months	-	14.4 (3.2 to 35.4)^C£^	475.7 ±194.8^A¥^	381 ± 301.8^A¥^	-	-	-
	6 months	-	-	421.8 ± 266.7^A¥^	319.3 ± 141.7^A¥^	-	-	-
	12 months	-	-	-	400.9 ± 391^A¥^	-	-	-
Adiponectin - ng/mL x 100 (blood serum)	Baseline	-	-	52.5 ± 36^A^	62.2 ± 43.2^A^	-	-	-
	3 months	-	-	49.1 ± 25.6^A^	70.7 ± 47.8^A^	-	-	-
	6 months	-	-	47.4 ± 34.3^A^	56.3 ± 34.6^A^	-	-	-
	12 months	-	-	-	71.1 ± 57.1^A^	-	-	-
Chemerin (gingival crevicular fluid)	Baseline	-	-	-	-	112.2 (107.9 up to 125.0)^D^	-	-
	6 weeks	-	-	-	-	47.6 (36.9 up to 53.8)^D^	-	-
Vaspin (gingival crevicular fluid)	Baseline	-	-	-	-	-	1.1 (0.8 up to 1.3)^D^	-
	6 weeks	-	-	-	-	-	0.5 (0.4 up to 0.6)^D^	-
Omentin-1 (gingival crevicular fluid)	Baseline	-	-	-	-	-	16.8 (15 up to 18.6)^D^	-
	6 weeks	-	-	-	-	-	25.7 (21.1 up to 31.9)^D^	-
**Systemic biomarkers of oxidative stress**								
8-hydroxy-deoxyguanosine (8-OHdG) - Pg/µg DNA (blood serum)	Before	-	-	-	-	-	-	1.9 ± 0.35^A^
	30 days	-	-	-	-	-	-	0.54 ± 0.23^A^
8-hydroxy-deoxyguanosine (8-OHdG) - pg/mL (saliva)	Before	-	-	-	-	-	-	927.94 ± 116.66^A^
	30 days	-	-	-	-	-	-	652.58 ± 139.51^A^
8-hydroxy-deoxyguanosine (8-OHdG) - pg/mL (gingival crevicular fluid)	Before	-	-	-	-	-	-	1178.44 ± 97.34^A^
	30 days	-	-	-	-	-	-	1003.66 ± 157.85^A^
Object of investigation	Follow-up	Taşdemir et al., 2016	Zuza et al., 2016	Martínez-Herrera et al., 2018a	Martínez-Herrera et al., 2018b	Çetiner et al., 2019	Peralta et al., 2020	Md Tahir et al., 2020
		( = 14)	( = 28)	( = 74)	( = 46)	( = 21)	( = 55)	( = 18)
**Blood hematological and biochemical index**								
Retinol-binding protein 4 (RBP4) - mg/L	Baseline	-	-	3.84 ± 1.06^A^	3.78 ± 1.11^A^	-	-	-
	3 months	-	-	3.46 ± 1.01^A^	3.44 ± 1.05^A^	-	-	-
Glucose - mg/L	Before	86.5 (80.5 to 92.2)^D^	99.8 ± 13.4^A^	95 ± 12^A^	95.2 ± 11.2^A^	-	-	-
	3 months	91.5 (78.5 to 96.5)^D^	102 ± 15.9^A^	94.8 ± 12^A^	95.7 ± 11.4^A^	-	-	-
	6 months	90.5 (78.5 to 97.5)^D^	-	-	-	-	-	-
Glycated hemoglobin (Hba1c) - %	Before	5.3 (5.2 to 5.6)^D^	5.4 ± 1^A^	-	-	-	-	-
	3 months	5.2 (5.1 to 5.4)^D^	4.4 ± 0.8^A^	-	-	-	-	-
	6 months	5.2 (5.1 to 5.5)^D^	-	-	-	-	-	-
Insulin - µU/mL	Before	18.3 (10.7 to 22.6)^D^	-	20 ± 14.6^A^	19.5 ± 10.9^A^	-	-	-
	3 months	14.7 (7.8 to 18.6)^D^	-	19.2 ± 11.3^A^	20.9 ± 11.9^A^	-	-	-
	6 months	17.1 (8.5 to 23.4)^D^	-	-	-	-	-	-
Homeostasis model assessment of insulin resistance (HOMA-IR)	Before	3.93 (2.25 to 5.06)^D^	-	4.73 ± 3.8^A^	4.58 ± 2.86^A^	-	-	-
	3 months	3 (1.8 to 4.18)^D^	-	4.61 ± 3.17^A^	5.04 ± 3.39^A^	-	-	-
	6 months	3.87 (2.05 to 5.37)^D^	-	-	-	-	-	-
Total cholesterol - mg/dL	Before	192.5 ± 31.4^A^	250 ± 14.1^A^	182 ± 34^A^	184 ± 33^A^	208 ± 34.6^A^	-	-
	3 months	200.2 ± 35.2^A^	210.6 ± 16.3^A^	185 ± 40^A^	188 ± 37^A^	200.3 ± 38.3^A^	-	-
	6 months	192.7 ± 37.6^A^	-	-	-	-	-	-
High density lipoprotein cholesterol (HDL) - mg/dL	Before	49.4 ± 15.6^A^	51.1 ± 3.5^A^	42 ± 11^A^	43.1 ± 11.4^A^	50.57 ± 9.52^A^	-	-
	3 months	46.5 ± 12.2^A^	50.4 ± 4.3^A^	44 ± 12^A^	43.8 ± 12.4^A^	55.14 ± 13.28^A^	-	-
	6 months	45.4 ± 14.1^A^	-	-	-	-	-	-
Low density lipoprotein cholesterol (LDL) - mg/dL	Before	102.6 ± 27.4^A^	170.8 ± 11.3^A^	121 ± 26^A^	116 ± 27^A^	128.64 ± 28.5^A^	-	-
	3 months	117.4 ± 34.4^A^	152.7 ± 14^A^	122 ± 31^A^	118 ± 29^A^	116.14 ± 38.7^A^	-	-
	6 months	111.4 ± 36.5^A^	-	-	-	-	-	-
Triglycerides - mg/dL	Before	166.4 (116.5 to 212)^C^	172.1 ± 14.2^A^	128 (98 to 167)^C^	126 (86 to 162)^C^	136.05 ± 46.2^A^	-	-
	3 months	157.9 (120 to 220.1)^C^	154.3 ± 15.9^A^	119 (98 to 152)^C^	132 (106 to 157)^C^	138.52 ± 51.64^A^	-	-
	6 months	167.5 (73 to 213.6)^C^	-	-	-	-	-	-
**Systemic biomarkers of inflammation**								
High-sensitivity C-reactive protein (hsCRP) - mg/L (blood serum))	Baseline	3.4 (3.4 to 5.4)^D^	3.75 ± 0.5^A^	8.33 ± 7.78^A^	4.33 (1.85 to 6.29)^C^	-	-	-
	3 months	3.3 (3.2 to 5.4)^D^	2.62. ± 0.2^A^	8.57 ± 7.92^A^	3.64 (1.62 to 6.32)^C^	-	-	-
	6 months	3.3 (3.2 to 8.2)^D^	-	-	-	-	-	-
Tumor necrosis factor-α (TNF-α) - pg/mL (blood serum)	Before	12.8 (10.5 to 15)^D^	-	17.23 ± 9.86^A^	19 ± 11.7^A^	-	-	-
	3 months	11.3 (7.2 to 16.1)^D^	-	13.9 ± 5.37^A^	14.4 ± 4.7^A^	-	-	-
	6 months	3.8 (2.6 to 6.4)^D^	-	-	-	-	-	-
	12 months	-	-	-	-	-	-	-
Tumor necrosis factor-α (TNF-α) - pg/mL (gingival crevicular fluid)	Before	-	-	-	-	9.0 ± 6.1^A^	-	-
	3 months	-	-	-	-	7.2 ± 5.9^A^	-	-
Interleukin (IL-6) - pg/mL (blood serum)	Before	2.23 (1.8 to 4.6)^D^	-	3.79 ± 2.04^A^	2.93 ± 1.31^A^	-	-	-
	3 months	2.13 (1.8 to 3)^D^	-	3.38 ± 2.48^A^	2.52 ± 1.44^A^	-	-	-
	6 months	2.04 (1.8 to 2.4)^D^	-	-	-	-	-	-
	12 months	-	-	-	-	-	-	-
Interleukin (IL-6) - pg/mL (gingival crevicular fluid)	Before	-	-	-	-	3.61 ± 4.43^A^	-	-
	3 months	-	-	-	-	1.76 ± 2.29^A^	-	-
Complement C3 (blood serum)	Before	-	-	-	128 ± 18^A^	-	-	-
	3 months	-	-	-	129 ± 28^A^	-	-	-
Visfatin - pg/mL (gingival crevicular fluid)	Before	-	-	-	-	21.53 ± 39.55^A^	-	-
	3 months	-	-	-	-	6.96 ± 3.49^A^	-	-
Resistin - ng/mL (blood serum)	Before	-	-	-	-	-	-	14.7 (10.8 to 18.5)^E^
	3 months	-	-	-	-	-	-	17.6 (12.4 to 22.7)^E^
Pentraxin-related protein 3 (PTX3) (blood serum)	Before	4.76 (3.1 to 7.9)^D^	-	-	-	-	-	-
	3 months	4.50 (3 to 6.9)^D^	-	-	-	-	-	-
	6 months	4.62 (3 to 6.8)^D^	-	-	-	-	-	-
**Periodontal pathogens count**								
(total bacterial count^†^ or x 10^6^ copy cells^§^)	Before	-	-	-	-	-	17.06 ± 4.62^F†^	1.7 (1.5 to 2)^E§^
	3 months	-	-	-	-	-	3.65 ± 1.22^F†^	1.5 (1.3 to 1.7)^E§^
	6 months	-	-	-	-	-	9.81 ± 2.81^F†^	-
	9 months	-	-	-	-	-	13.88 ± 5.49^F†^	-
(total bacterial count^†^ or x 10^6^ copy cells^§^)	Before	-	-	-	-	-	58.52 ± 18.77^F†^	0.6 (0.4 to 0.8)^E§^
	3 months	-	-	-	-	-	22.05 ± 7.2^F†^	0.7 (0.5 to 0.9)^E§^
	6 months	-	-	-	-	-	55.87 ± 16.24^F†^	-
	9 months	-	-	-	-	-	33.24 ± 8.79^F†^	-
(x 10^6^ copy cells)	Before	-	-	-	-	-	-	1.0 (0.7 to 1.3)^E^
	3 months	-	-	-	-	-	-	0.6 (0.4 to 0.9)^E^
	6 months	-	-	-	-	-	-	-
	9 months	-	-	-	-	-	-	-
(total bacterial count)	Before	-	-	-	-	-	18.14 ± 6.01^F^	-
	3 months	-	-	-	-	-	5.64 ± 1.51^F^	-
	6 months	-	-	-	-	-	14.77 ± 4.09^F^	-
	9 months	-	-	-	-	-	7.14 ± 2.32^F^	-
(total bacterial count)	Before	-	-	-	-	-	92.0 ± 20.7^F^	-
	3 months	-	-	-	-	-	65.6 ± 36.6^F^	-
	6 months	-	-	-	-	-	48.2± 15.4^F^	-
	9 months	-	-	-	-	-	21.1 ± 6.6^F^	-
**Quality of Life**								
OHqOL	Baseline	-	-	-	-	-	-	-
	6 months	-	-	-	-	-	-	-
OIDP	Baseline	-	-	-	-	-	-	-
	6 months	-	-	-	-	-	-	-

BAS, before-and-after (pre-post) studies, corresponding to data from intervention group of patients with obesity; *n*, sample size; TNF-α, tumor necrosis factor alpha; IL-6, interleukin-6; OHQoL, oral-related health quality of life; D, mean difference from 2 months follow-up to baseline; ^A^, mean ± standard deviation; ^C^, median (25 to 75 percentil); ^D^, median (minimum to maximum); ^E^, median (95 % confidence interval); ^F^, number ± standard error; -, variable not assessed by the authors; NA, data not available.

### Results of syntheses

#### RCT studies

The clinical approach performed in RCT studies were NSPT plus antibiotic therapy,^36^ and NSPT in the IG and no-PT in the CG^4,35^. Two studies^4, 35^ used 0.12% chlorhexidine, and one^36^ study used 0.12% chlorhexidine and 0.05% cetylpyridinium chloride twice daily for 14 days post-therapy.

#### Adipokines

In the study by Akram et al.,^4^ the mean resistin level differed between the CG and IG (14.25 ± 4.58 ng/mL and 12.26 ± 1.24 ng/mL, respectively; p < 0.05). There was a significant reduction in resistin after NSPT (p < 0.05) in the IG but not in the CG (mean difference 0.65 ± 1.24 ng/mL and 0.78 ± 4.08 ng/mL, respectively) – logistic regression analysis revealed that change in salivary resistin level was not significantly associated with improvement in probing depth (PD) or clinical attachment level (CAL), even after smoking control (p > 0.05). According to the authors, resistin level did not differ between the CG and IG at the 12-week follow-up.

#### Quality of life

Basher et al.^35^ reported a decrease in Oral Health Impact Profile-14 (OHIP-14) PI and OHIP-14 EI and an increase in OHIP-14 SS over time in both groups (p < 0.05). The mean OHIP-14 EI at 12 weeks post-NSPT decreased in both CG and IG at 0.65 (1.02%) and 0.47 (0.91%), respectively. Only “bad breath” (functional limitation domain) and “food impaction” (psychological discomfort domain) were significantly reduced (p < 0.05).^35^ According to the authors, quality of life did not differ between the CG and IG.

#### Subset analysis - non-surgical periodontal therapy plus antibiotic therapy

Montero et al.^36^performed a study associating NSPT with antibiotic therapy. The test group (IG) received an intensive periodontal treatment with two sessions of non-surgical subgingival instrumentation and administration of azithromycin 500 mg q.d. for three days, administered during the last NSPT session. The control group (CG) received minimal periodontal treatment, which consisted of two sessions of supragingival plaque and calculus mechanical removal and administration of placebo medication for 3 days. Both groups received an antiseptic mouth rinse containing 0.12% chlorhexidine and 0.05% cetylpyridinium chloride and oral hygiene instructions.

#### Blood pressure

Systolic blood pressure (SBP) was significantly reduced at 3 months of follow-up in the IG compared with the CG after adjustment for covariates [7.3mmHg (95%CI: 1.9–12.6; p = 0.008)]. The reduction in diastolic blood pressure (DBP) in the IG lasted six months after NSPT: i- 3 months of follow-up: 7.8mmHg (95%CI: 1.3–14.4; p = 0.019); and ii- 6 months of follow-up: 11 mmHg (95%CI: 2.9–19.1; p = 0.009).^36^ According to the authors, no other metabolic, vascular, and renal parameters showed any significant difference.

#### Hematological and biochemical index

Three months after NSPT, glycated hemoglobin (HbA1c) decreased in the IG compared with CG – difference adjusted for covariates, 0.3% (95%CI: 0.1–0.6; p = 0.013). The proportion of patients with HbA1c ≥ 7% decreased significantly in the IG, from 31.25% at baseline to 18.8% at 3 months of follow-up (p = 0.028), with no changes in the CG (post-hoc analyses); no differences between the two groups were observed six months after NSPT. The multilevel linear regression determined that the variance in HbA1c was only predicted by being in the IG (p = 0.013) and by the baseline HbA1c percentage (p < 0.001), without any significant additional effect in the model for age, sex, BMI, or smoking status. In addition, no differences between the CG and the IG were observed for white blood cell count, fibrinogen, and α-1 antitrypsin at any time point after therapy.^36^

The authors^36^ reported a decrease in the mean high-sensitive C-reactive protein (hsCRP) concentration after three and six months in the IG, but not in the CG. The difference between groups, adjusted for age, sex, smoking, baseline BMI, and hsCRP was 1.4 mg/L (95%CI: 0.5–2.2; *p* = 0.001) at three months and 1.2 mg/L (95%CI: 0.4–2.0; *p* = 0.004) at 6 months of follow-up. The odds ratio for IG versus CG from an hsCRP value ≥ 3 to < 3mg/L was 5.4 (95%CI: 1.0–31.6; *p* = 0.040). 68.8% of patients in the IG experienced a reduction in hsCRP levels within 6 months of follow-up, while this percentage was 29% in the CG (*p* < 0.001). The NSPT led to a 30.8% reduction in hsCRP from baseline and a difference of 1.2 mg/L at 6 months of follow-up compared to the CG. Improvements in periodontal health, despite actively following strict cardiovascular risk reduction protocols, significantly improved hsCRP levels and cardiovascular risk. In the multilevel linear regression, baseline hsCRP levels (p < 0.001) and smoking (*p* = 0.014) significantly and independently predicted the variance of hsCRP decline over six months in the IG.

#### Cytokines

Montero et al.^36^also reported a significant decrease in IL-1β and TNF-α at 3 months of follow-up in the IG compared with the CG. However, no differences between the groups were observed for these biomarkers at 6 months of follow-up, or for IL-6 and IL-8 at any time point after therapy.

#### Microbiological evaluation

The authors^36^ reported counts of anaerobic bacteria and high proportions and counts of *Porphyromonas gingivalis* (*Pg*) in all patients at baseline. The NSPT significantly reduced both the counts of anaerobic bacteria and *Pg*, and this microbiological impact was associated with significant reductions in hsCRP.

## BAS studies

### Hematological and biochemical index

There was significant improvement in anthropometric and metabolic parameters and C3 (immunity) 12 weeks after NSPT in the obesity diet group (*p* < 0.05).^46^ Altay et al.^37^ reported a significant reduction in serum levels of HOMA-IR score and Martínez-Herrera et al.^46^ reported a significant decrease in RBP4 three months after NSPT.

Al Zahrani et al.^11^reported a mean difference in hsCRP of 0.19 ± 0.32 (*p* = 0.015). According to Zuza et al.,^44^ patients with obesity and periodontitis who received basic PT exhibited significant reduction in the serological levels of total cholesterol, low-density lipoprotein, triglycerides, and hsCRP 90 days after NSPT. In contrast, Altay et al.,^37^ Taşdemir et al.,^43^ and Martínez-Herrera et al.^46,47^ reported a non-significant reduction in hsCRP after NSPT.

### Cytokines

Altay et al.,^37^ Taşdemir et al.,^43^ and Martínez-Herrera et al.^46,47^ reported a decrease in serum TNF-a levels after NSPT. Balli et al.^41^ reported the same result for GCF. According to Gonçalves et al.,^39^ concentrations of TNF-a and leptin increased in shallow and deep sites of patients with obesity at 6- and 12 months of follow-up compared to baseline (p < 0.05). There were no statistically significant changes in the GCF levels of IL-6 and resistin, and in the serum levels of any adipokines at any time point after therapy. In contrast, Çetiner et al.^45^ did not observe a significant decrease in TNF-α in the GCF after NSPT. Furthermore, serum PTX-3 levels were not significantly reduced after NSPT.^43^

Seven studies evaluated the concentration of IL-6 in serum^37,43,45,46,47^ and in the GCF.^38,39,40^ Only two studies (2:5 ratio, 28.57 %) reported significant reductions in serum and GCF IL-6^37,40^(respectively).

### Adipokines

Altay et al.^37^ reported a reduction in serum leptin levels after NSPT, but Gonçalves et al.^38^ reported no changes in serum leptin levels three and six months after NSPT. Periodontal therapy reduced the GCF levels of chemerin, vaspin, omentin-1, and visfatin in the GCF^40,41,45^ and increased leptin levels in the shallow and deep sites 12 months after therapy, compared to baseline^39^ (*p* < 0.05). There were no statistically significant changes in the serum and GCF levels of resistin and adiponectin at any time point after NSPT.^38,39,49^

### Oxidative stress

Levels of 8-OHdG in plasma, saliva, and GCF significantly decreased after NSPT (p < 0.01).^42^

### Microbiological evaluation

Within nine months, *Pg* and *Aggregatibacter actinomycetemcomitans* (*Aa*) significantly decreased.^48^ Small counts of *Tannerella forsythia* (*Tf*) were observed only at 3 months of follow-up; however, reductions in *Tf* count were not maintained at 9 months of follow-up. NSPT also reduced *Treponema denticola* (Td) count (p < 0.05). In contrast, Md Tahir et al.^49^ reported no significant changes in mean *Pg* and *Tf* counts at 12 weeks of follow-up. According to the authors, the mean *Prevotella intermedia* (*Pi*) count decreased by almost half 12 weeks after NSPT.

### Quality of life

OHRQoL (oral health-related quality of life) increased and OIDP (oral impact on daily performance) decreased six months after NSPT (p < 0.05). Regarding OIDP, pain, discomfort, and functional limitation significantly improved at 6 months of follow-up. The prevalence of oral impacts on activities of daily living, such as eating and enjoying food and cleaning teeth, significantly decreased six months after NSPT.^50^

### Risk of bias in studies

All RCTs included in this review were considered to have a low risk of bias^4,35,36^ (Figure 4A). Nine BAS studies were classified as low risk of bias.^11,38,39,40,41,42,43,44,45^ Confounding and missing data domains accounted for the low methodological quality of the BAS studies [serious risk of bias^37,46,47,50^ and critical risk of bias^48,49^(Figure 4B)].

### Certainty of evidence

Regardless of the variation in the approach of the applied intervention, outcome assessed, type of sample, and evaluation time, the overall certainty of the evidence ranged from moderate (evidence from RCT) to low or very low (evidence from BAS studies). Evidence from RCT was seriously affected by the imprecision item due to the small number of individuals included in the syntheses. It is important to mention that some aspects such as the precision of the estimates and consistency of the results could not be evaluated because a meta-analysis was not performed and the syntheses for all outcomes always included a single RCT, respectively. On the other hand, evidence from BAS studies was seriously or very seriously affected by the risk of bias item, seriously affected by the inconsistency item in most of the syntheses that included more than one study, and seriously affected by the imprecision item due to the insufficient number of participants evaluated.

## Discussion

Recognizing the limited number of studies on the subject and analyzing the results of this study with caution, the available data support at least moderate evidence on the benefits of NSPT for cardiometabolic, inflammatory, and microbiological parameters in patients with obesity and periodontitis, based on RCT studies. As expected, the certainty of evidence from BAS studies was limited by the study design. Pre-post analysis showed local, systemic, and quality of life improvement after subgingival instrumentation of periodontal pockets, corroborating the findings of RCT studies.

Periodontitis, as an inflammatory disease, is linked to non-communicable chronic diseases, such as obesity. A possible mechanism that contributes to this relationship may be the low-grade systemic inflammation caused by periodontitis, which is common in many chronic conditions. In contrast, systemic diseases also affect periodontitis.^52^

NSPT can significantly reduce several biochemical markers of obesity and provide periodontal clinical improvements, but these are smaller than in non-obese individuals.^53^

The significant reduction in SBP and DBP three months after effective PT^36^ corroborated the meta-analysis by Muñoz Aguilera et al.^54^ Law et al.^55^ associated PT with 10-mmHg reduction in SBP or a 5-mmHg reduction in DBP, and 25% to 30% reduction of cardiovascular events. This can be considered a significant benefit, especially due to suboptimal adherence to pharmacotherapy for hypertension.^56,57,58,59^ The serum levels of total cholesterol, LDL, and triglycerides also improved after NSPT, compared to baseline.^44^ Periodontal therapy was effective in reducing HbA1c and blood pressure at 3 months of follow-up, suggesting early benefits of periodontitis treatment for metabolic control and vascular function.^36,60,61^ The lack of repeated periodontal interventions during the study^36^ appears to explain the late reversal of HbA1c improvement.^62,63^

Non-surgical periodontal therapy led to a 30.8% decrease in hsCRP from baseline values and showed a difference of 1.2 mg/L at 6 months of follow-up compared with the CG,^36^ reducing cardiovascular risk.^64,65^ On the other hand, the results from the PAVE study suggest that PT is not able to maintain the reduction of serum hsCRP levels at 6 months of follow-up.^66^ In addition, BAS studies showed significant^11,44^ or non-significant^37,43,46,47^ reduction in serum hsCRP levels after NSPT.

Most studies address the effects of obesity on the periodontium,^4,12,67,68^ but the literature remains scarce on the benefits of periodontitis therapy for patients with obesity. One RCT reported a significant decrease in serum levels of IL-1β and TNF-α at 3 months of follow-up in the IG, and no difference for IL-1β, IL-6, IL-8, and TNF-α six months after PT.^36^ The BAS analyses showed a decrease in TNF-α levels in both serum and GCF matrices.^37,41,43,46,47^ Contrasting results such as no significant change in GCF levels of IL-6, TNF-α, and resistin, and in the serum levels of any adipokines at any time point after therapy,^39,43,45^ reinforce the need for more RCTs on the subject.

NSPT improved the circulating levels of proinflammatory cytokines and C3 and insulin resistance, compared to baseline.^37,46^ According to Akram et al.,^69^ GCF may be more sensitive than saliva to detect changes in cytokine levels caused by local inflammation. The authors reported a significant reduction in resistin after NSPT in the IG but not in the CG – this result was not correlated with improvement in PD or CAL, probably because only shallow and moderate sites improved and there were higher resistin levels in the CG than in the IG at baseline, thus introducing a risk of bias. Despite the inclusion of smokers is considered an important potential confounding factor,^70-72^ there was no significant impact on periodontal outcomes in the study by Akram et al.^69^ Other studies have associated NSPT with decreased levels of resistin in the saliva, suggesting the need for further studies on this biomarker.^67,73^

Increased inflammatory factors, disturbances in glycolipid metabolism, and adipokine overexpression in obesity can be worsened by periodontitis.^74^ Although serum and GCF concentrations of leptin, adiponectin, and oxidative stress biomarkers remain uncertain and underexplored in the obesity-periodontitis scenario, chemerin, vaspin, omentin-1, and visfatin in GCF improved after NSPT.^40,41,45^ In the study by Öngöz Dede et al.,^42^ 8-OHdG, a powerful periodontal disease marker,^75^ significantly decreased in plasma, saliva, and GCF after NSPT.

The improvement of the proinflammatory state represents a huge benefit of periodontal therapy for patients with obesity, as it interferes with insulin resistance and metabolic disorders, hepatic steatosis, and cardiovascular diseases.^76-84^ Subcutaneous and visceral adiposity, CRP, and IL-6 also represent a risk for type 2 DM.^85,86^

People with obesity have higher rates of periodontal pathogens and an increased risk of progressive attachment loss than normal-weight individuals.^87^ Mean *Pg, Tf*, and *Pi* counts can be reduced by 7% to 45% 12 weeks after periodontitis therapy^88,89^ and ranged from 18% to 99% after NSPT in patients with DM.^90,91^ Periodontal therapy appears to reduce *Pg, Pi, Aa, Tf*, and Td counts for three months,^48,49^ although Md Tahir et al.^49^ reported no significant changes in mean *Pg* and *Tf* counts at 12 weeks of follow-up. Diagnostic criteria for periodontitis, the full-mouth disinfection protocol,^92,93^ and the periodontal maintenance phase^94-96^ adopted by Peralta et al.^48^ may explain this divergence. The SRP strategy with and without chlorhexidine should not respond to this difference,^97,98^ although the difference between the response to one-stage full-mouth therapy and quadrant-by-quadrant root planing could be expected.^99^ Montero et al.^36^ adopted azithromycin as an adjuvant antibiotic for NSPT and used 0.12% chlorhexidine and 0.05% cetylpyridinium chloride twice daily for 14 days post-therapy. The authors reported a decrease in anaerobic bacteria and *Pg* counts after PT and associated this result with significant reductions in hsCRP.

In our inclusion criteria, we accepted all types of treatment with a subgingival approach, including adjunctive antimicrobials, photodynamic therapy, laser therapy, and surgical treatment.

The results presented by Monteiro et al^36^, whose study used antibiotics, demonstrated improvement in parameters related to hsPCR, IL-1β, TNF-α, and P. gingivalis count. These results were similar to those found in the BAS studies reported by Zuza et al.,^44^ Altay et al.,^37^ Tasdemir et al.,^43^ Martin-Herrera et al.,^46,47^ and Md Tahir et al., ^49^ which did not use antibiotics. Therefore, there was no advantage in these results when compared with the other studies included in this review. Regarding the other parameters evaluated in the study by Monteiro et al.,^36^ there was no difference between the control and test groups. Adjuvant antibiotic therapy does not seem to have caused significant changes when compared with studies that did not use such therapy.

Interestingly, the immunomodulatory properties of azithromycin for the levels of cytokines and chemokines should be considered confounding factors by the author.^100^ Macrolides decrease the formation of proinflammatory cytokines, adhesion molecules, reaction to chemoattractants, oxidative burst, and adaptive immunity, and promote the release of anti-inflammatory cytokines, neutrophil apoptosis, and neutrophil degranulation.^100,101^ The pharmacological effects of azithromycin on the various cytokines are very complex and are dependent on dose, targeted cell, and temporal differences in terms of host modulatory function.^102-107^ Therefore, the cardiovascular effect reported by Montero et al.^36^ could be attributed to antibiotics rather than to PT. In addition, although hCRP is a surrogate biomarker for cardiovascular risk,^108,109^ its predictive value may be limited.^110^

Commensal microbiota and complement are both necessary for *Pg*-induced bone loss, as confirmed in germ-free or C3a- and C5a receptor-deficient mice inoculated with Pg. Regarding the pathogenicity of key species in periodontitis, *Pg* was able to subvert complement receptor 3 and anaphylatoxin C5a receptor signaling. Even a single low-abundance species can disrupt homeostasis, leading to dysbiosis, inflammatory events, and disease. In this context, effective periodontal therapy should require activation of the inductive or effector pathways of the complement.^111-113^ The improvement in *Pg* counts and serum levels of C3 reported in the studies may be correlated.

The slow and progressive nature of chronic periodontitis allows the patient to adapt to clinical symptoms and seek dental care later. Therefore, limited perception of patients to recognize chronic periodontitis as a condition may affect the OHRQoL – multidimensional construct that includes a subjective evaluation of the individual’s oral health, functional well-being, emotional well-being, expectations, and satisfaction with care and sense of self.^35,114^ The effects of PT on quality of life were inconclusive due to the limited number of studies and the subjectivity of the method.

The present systematic review has several limitations, and the results must be interpreted with caution. The included studies used different criteria to define periodontitis and obesity, different periodontal therapy protocols, periodontal maintenance phase, objects of investigation, and low number of included participants.

To strengthen the quality of this systematic review, no restrictions were applied to databases, records, and other sources in the screening process. The search and selection of articles, data collection, and synthesis were performed independently by two researchers and a validated quality assessment tool was used. The certainty of evidence was also evaluated following the GRADE approach. Publication bias cannot be excluded, as studies with positive results tend to be more easily published.^115^ Performing a meta-analysis was considered, but the limitations described above make it difficult. Despite the methodological heterogeneity and scarcity of publications on the subject, some clear pattern was established.

Based on the limitations found during this systematic review, future controlled and well-planned clinical trials are needed to evidence the benefits of periodontal therapy on systemic parameters in patients with obesity and periodontitis.

## Conclusion

The current findings suggest that periodontitis therapy has the potential to improve blood pressure, serum levels of total cholesterol, LDL, triglycerides, HbA1c, insulin resistance, hsCRP, IL-1β, TNF-α and C3, GCF levels of TNF-α, chemerin, vaspin, omentin-1, visfatin and 8-OHdG, and *Pg, Pi, Aa, Tf*, and Td counts.

The benefits reported in this review were achieved with non-surgical periodontal therapy, and only one study used an adjuvant antibiotic, having obtained equivalent results to those of the other studies.

Future well-designed studies are important to elucidate the impact and benefits of periodontal therapy on hematological and biochemical index, biomarkers of inflammation and oxidative stress, quality of life, and periodontal pathogen counts in patients with obesity and periodontitis.
